# Influence of the Umbilical Cord Insertion Site on the Optimal Individual Birth Weight Achievement

**DOI:** 10.1155/2014/341251

**Published:** 2014-05-25

**Authors:** Sophie Brouillet, Anaïs Dufour, Fabien Prot, Jean-Jacques Feige, Véronique Equy, Nadia Alfaidy, Pierre Gillois, Pascale Hoffmann

**Affiliations:** ^1^Gynaecology, Obstetric and Reproductive Medicine Department, Grenoble University Hospital, BP 217, 38043 Grenoble Cedex, France; ^2^Commissariat à l'Energie Atomique (CEA), DSV-iRTSV, 38043 Grenoble Cedex, France; ^3^Université Grenoble-Alpes, 38041 Grenoble, France; ^4^Institut National de la Santé et de la Recherche Médicale U1036 (INSERM U1036), Laboratoire Biologie du Cancer et de l'Infection (BCI), Laboratoire BCI-iRTSV, CEA Grenoble, 17 rue des Martyrs, 38054 Grenoble Cedex 9, France; ^5^UJF-Grenoble 1, CNRS, TIMC-IMAG UMR 5525, Themas, 38041 Grenoble, France

## Abstract

*Study Question*. To determine whether the umbilical cord insertion site of singleton pregnancies could be linked to the newborn birth weight at term and its individual growth potential achievement. *Material and Methods*. A cohort study including 528 records of term neonates was performed. Each neonate was assessed for growth adjusted for gestational age according to the infant's growth potential using the AUDIPOG module. We considered two categories of umbilical cord insertions: central and peripheral. Intrauterine growth restriction was defined as birth weight below the 10th percentile. Statistical analysis was performed using Chi-square, Student's *t* test, Wilcoxon test, ANOVA, and logistic regression. *Results*. We observed a total of 343 centrally inserted cords versus 185 peripheral cords. There were twice as many smokers in the mothers of the peripheral category compared to the centrally inserted ones. More importantly, we demonstrated that only 17/343 (5.0%) of infants with central cord insertion were growth restricted, compared to 37/185 (20.0%) of the infants born with a peripheral insertion. Neonates with centrally inserted cord were significantly heavier. *Conclusion*. The umbilical cord insertion site of singleton pregnancies is associated with the newborn's birth weight at term and its individual growth potential achievement.

## 1. Introduction 

Fetal growth is characterized by its genetically predetermined growth potential; however, it is further modulated by maternal, fetal, placental, and environmental factors [1, 2]. Hostile circumstances can threat the achievement of the growth potential of the fetus throughout pregnancy [2]. Limitations of fetal growth potential are known to lead to a variety of adverse perinatal and adult conditions, ranging from stillbirth to chronic diseases in adulthood [3–5]. Adequate growth of the fetus throughout pregnancy is highly dependent on the normal development of the umbilical cord (UC), a structure that connects the growing embryo to its placenta by the third week of gestation [6, 7]. The UC ensures oxygen and nutrient supplies to the fetus throughout pregnancy. During the last decades, numerous studies have highlighted that UC insertion site could be relevant in obstetrics as its abnormal positioning has been associated with numerous complications of pregnancy, including preterm delivery, low birth weight, growth restriction, stillbirth, increased rate of emergency caesarean section, and low Apgar scores [7–12].

The UC insertion site can be subdivided into four categories: central, paracentral, marginal/battledore, and velamentous/membranous. The central/paracentral category is considered as the normal condition. It is well accepted that UC insertion is considered aberrant when attached at the edge of the placental disk (marginal/battledore) or when it is inserted into the chorioamniotic membranes (velamentous/membranous), which often leads to fetal death [8, 13–15]. Recent reports suggested that a peripherally inserted UC might also be associated with pregnancy pathologies [16]. Although noncentral cord insertion is more frequent in twin pregnancies, singletons are also concerned with an approximate frequency of 7% for marginal and 1% for velamentous insertion [17].

The etiology of noncentral cord insertion is not clear and it is probably influenced by many factors. Three theories have been proposed: first is the “blastocyst polarity” theory, which hypothesizes that aberrant insertion site results from malpositioning of the blastocyst during implantation, with consequent defective placental disk orientation [18]; the second is the “abnormal placental development because of decreased chorionic vessel branching” theory, which posits that noncentral insertion results from abnormal vasculogenesis in the placenta [19–21]; and the third one is the “trophotropism/placental migration” theory, which proposes that aberrant insertion develops later on during pregnancy when the placenta migrates toward sites of optimal perfusion [8]. Because of the early appearance of abnormal cord insertion during pregnancy, the latter theory has been excluded [20, 22].

Noncentral cord insertion is closely associated with early impaired development and function of the placenta, suggesting genetic and/or environmental associated mechanisms [6, 23]. Fetal growth is dependent on oxygen and nutrient-transfer capacity of the placenta, which is highly associated with the vascular network development within the chorionic villi. Abnormal umbilical cord insertion is associated with smaller placenta [23] and lower placental vessel density [24]. The placental insufficiency accompanying abnormal cord insertion may increase the susceptibility to perinatal risk often associated with these conditions [25, 26]. We thus hypothesize that optimal placentation will result in a central insertion of the umbilical cord which in turn allows an optimal growth of the fetus throughout gestation. Therefore, the aim of the present study was to examine if the site of umbilical cord insertion within the placenta of singleton pregnancies could be correlated to the newborn birth weight at term and to its individual growth potential achievement.

## 2. Material and Methods

**Figure 1:**
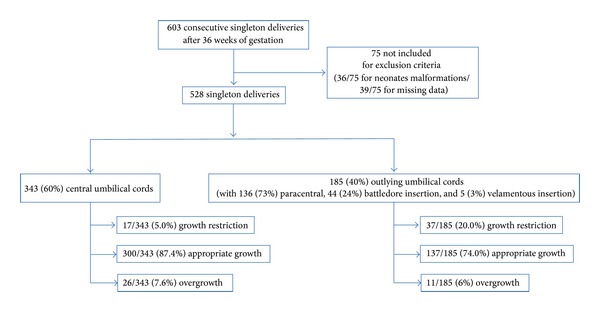
Flowchart of the study group.

A cohort study, including 603 consecutive singleton deliveries after 36 weeks of gestation (WG), from August 1, 2006, to December 31, 2006, was performed at the Grenoble University Hospital, Maternity Clinic. Neonates with malformations (36/75) and incomplete files (39/75) were excluded (*n* = 75, 12.4%). A total of 528 files were evaluated (Figure 1). The data were then collected from the computerized files. The maternal data collected included ethnicity (Caucasian, North African, African, or Asian), age, prepregnancy body mass index (BMI), tobacco use, spontaneous first trimester pregnancy loss records, uterine anomalies such as myomas, and prepregnancy health status influencing fetal growth (i.e., hypertension, autoimmune diseases, cardiomyopathy, diabetes, renal impairment, parity, and pregnancy pathology, such as gestational diabetes, preeclampsia/eclampsia, anaemia, and obstetric cholestasis). The selected neonatal items were as follows: gestational age at birth (in days), baby's gender, size, and weight, and cord insertion site. Two categories were used: central insertion and peripheral insertion (paracentral, battledore, and velamentous). Central UC was defined as UC insertion near the center of the placenta (i.e., less than 3 cm from the center). Paracentral UC was defined as insertion of the UC more than 3 cm from the center and more than 2 cm from the nearest margin. Marginal UC insertion was defined within 2 cm of the disc's edge, whereas velamentous insertion represents an UC insertion directly into the membranes. Each new born was individually assessed for growth and adjusted to its gestational age according to the infant's growth potential. This was performed based on AUDIPOG online fetal growth assessment module (AUDIPOG—Association of Users of Computerized Medical Records in Paediatrics, Obstetrics and Gynaecology—http://www.audipog.net/) [27, 28]. This French module takes into account maternal age, prepregnancy BMI, birth rank, newborn gender, gestational age, birth weight, and length to predict the individual infant's genetic growth potential [27–30]. From a sample of 43,654 infants from the AUDIPOG database, two statistical models gave individualized limits of birth weight and birth length below which, after adjustment for its individual growth potential, a newborn must be considered as FGR in weight and/or in length. This new approach has been validated in several publications [27–30] and allows classifying some newborns as “constitutionally small” due to their low growth potential and identifying newborns with fetal growth restriction (FGR). In this study, we used the individualized limits of birth weight to identify newborns with FGR. Once the infant's estimated personal percentile is calculated with AUDIPOG curves, each neonate is classified under three categories: growth restricted (less than the 10th percentile, with severe growth restriction below the 3rd percentile), appropriate for gestational age (from the 10th percentile to the 90th percentile), or large for gestational age (over the 90th percentile).

Variables were described as mean and standard deviation for continue quantitative data, number and proportion for qualitative data, and median and interquartile range for discrete quantitative data. The normality was checked with histogram of the sample data, also with skewness and kurtosis standardized moments (age and size) and with the Shapiro Wilk normality test (birth weight). The nonnormal initial distribution (BMI and weight) was transformed with a log function. The analyses with nonnormal distribution (parity) were switched to nonparametric test, as Wilcoxon test. Student's *t*-test was appropriate for categorical and continuous data. Chi-square test was appropriate for categorical data. We performed multivariate ANOVA (umbilical cord insertion site and tobacco) and logistic regression modeling for fetal growth restriction explained by tobacco and umbilical cord insertion site in the model, both of them with interaction. The OR and 95 IC for each factor were performed.

A *P* value <0.05 was considered significant. The statistical analyses were performed using the R-software (V3.10).

## 3. Results

The flowchart of the study group is represented in Figure 1. Analyses of our cohort showed that 343/528 (65.0%) of umbilical cords were centrally inserted, whereas 185/528 (35.0%) were peripheral (Table 1). Within the peripherally inserted umbilical cord, paracentral insertion was observed in 136/185 (73.5%) UC, battledore insertion in 44/185 (23.8%) UC, and velamentous insertion in 5/185 (2.7%) UC (Table 1). The main demographic characteristics of the two populations are summarized in Table 2. There was no significant difference between the two groups in terms of age, parity, BMI, or ethnicity of the mother.

Confounding factors traditionally associated with FGR are presented in Table 3. No significant difference between the two groups was observed when comparing prepregnancy pathologies, uterine anomalies, and first trimester spontaneous pregnancy losses. Interestingly, there were twice as many smokers in the mothers of the peripheral category. There were 34/343 (9.9%) smokers in the mothers of the central cord inserted group and 37/185 (20.0%) in the peripheral group, respectively (Chi2 = 10.5, df = 1, *P* < 0.01). No difference was observed between the two groups when comparing the occurrence of main pregnancy pathologies, such as diabetes, preeclampsia, anaemia, and obstetric cholestasis.

The detailed infant characteristics are shown in Table 4. There was no difference in the gestational age at birth between the two categories (one-way ANOVA = 3, Df = 1, *P* value = 0.053). Our analysis demonstrated that the mean neonatal weight was statistically different between the two categories (*t*-test, *P* < 0.001). Interestingly, the infants born with a centrally inserted cord were 235 g heavier. When analyzing the achievement of their individual growth potential, we found that only 17/343 (5.0%) of infants with central cord insertion were under the 10th percentile (with 5/343 (1.5%) under the 3rd percentile and 12/343 (3.5%) between the 3rd and the 10th percentile, resp.), compared to 37/185 (20.0%) in the peripheral group (with 15/185 (8.1%) under the 3rd percentile and 22/185 (11.9%) between the 3rd and the 10th percentile, resp.). Overall, peripheral UC was strongly associated with growth restriction (Chi-square, *P* < 0.001).

We performed a multivariate analysis and a logistic regression analysis to determine whether smoking and peripheral insertion contribute to FGR (Table 5). ANOVA analysis did not show any significant interaction effect between tobacco and umbilical cord insertion site (interaction factor, *F* value = 0.39, df = 1, *P* = 0.53). The tobacco factor was nonsignificant (*F* value = 1.92, df = 1; *P* = 0.16). However, we found a strong interaction between peripheral UC insertion site and FGR (*F* value = 37.874, df = 1; *P* = 1.5 × 10^−9^). In accordance with these data, the logistic regression confirms the significant interaction between peripheral umbilical cord insertion site and fetal growth restriction (Table 5). Compared to central UC insertion site, the odds ratio of having a growth restricted newborn increased to 4.49 (95% confidence interval 2.26 to 8.89) for peripheral UC insertion site. There was no significant interaction between tobacco and FGR (OR [95% CI] = 0.97 [0.20–4.53]) or between tobacco and peripheral cord insertion (OR [95% CI] = 2.03 [0.55–7.48]).

## 4. Discussion

In the present study we demonstrate a significant difference in the infant birth weight at term between central and peripheral umbilical cord insertion according to the individual growth potential. Our study aimed to investigate an old dogma regarding the relationship between cord insertion and infants' outcomes. The percentages of centrally and aberrant inserted cords reported in our study represent an intermediate finding between two previous reports. The first study, by Rolschau [31], reported only 2.68% (*n* = 12, 95% CI = 1.1–4.30%) of battledore insertion and 1.15% (*n* = 5, 95% CI = 0.3–2%) of velamentous insertions in 447 singleton pregnancies. In 2013, the group of Rasmussen reported 6.3% (*n* = 39,403; 95% CI = 6.3–6.4%) of battledore insertion and 1.5% (*n* = 9,500; 95% CI = 1.5–1.6%) of velamentous insertions in 623,478 singleton pregnancies [23]. We observed 8.3% (*n* = 44; 95% CI = 5–16%) of battledore and 0.95% (*n* = 5; 95% CI = 0–9%) of velamentous insertions in 528 singleton pregnancies. These results are closer to the study of Rasmussen's group. The discrepancies observed with the first study could be explained by several hypotheses. Compared to the data from Rolschau [31] published in the 1970s, the higher percentage of battledore insertion observed in our study might be due to the increasing incidence of several factors that have been shown to affect pregnancy outcomes [32, 33], such as inadequate maternal environment (e.g., increasing maternal age of conception [34, 35], maternal smoking, and stress [36–38]), multiple births [37], pregnancies conceived with the aid of assisted reproductive technology [39], endocrine disruptors [40, 41], and specific environmental parameters (e.g., air pollution) [42].

We observed that tobacco use was twice as frequent in the peripheral inserted cord group compared to the normal one. This is in accordance with previous studies from Thirkill et al. [43] and a recent study from Holloway et al. [44] demonstrating that the cigarette smoke and nicotine inhibited placental endothelial cells responses as well as the migration of the trophoblast cells. Moreover, Shiverick and Salafia reported in a recent report that nicotine and polyaromatic hydrocarbons disturbed trophoblast cell proliferation and invasion [45]. More recently, Zdravkovic et al. have demonstrated that nicotine downregulated the l-selectin system and consequently inhibits cytotrophoblast migration from the cell columns [46]. Tobacco might then be an early inhibitor of trophoblastic shell formation. In 2013, the group of Rasmussen reported that smoking early in pregnancy slightly increased the risk of velamentous cord insertion (OR = 1.2, 95% CI = 1.1–1.3) but not marginal insertion (OR = 0.99, 95% CI = 0.96–1.01) [23]. In our study, maternal smoking during pregnancy did not significantly increase the risk of peripheral umbilical cord insertion site or FGR. This result was unexpected as it is well established that tobacco consumption during pregnancy leads to a reduction in birth weight and that smoking cessation prior to the third trimester results in a reduction in the risk of fetal growth restriction [36–38]. The relatively small number of patients in our study could explain the discrepancy in these results. Indeed, one can speculate that larger studies might uncover weaker associations such as tobacco/FGR and tobacco/peripheral UC insertion site. Further studies are required to determine whether maternal smoking during pregnancy is associated with the occurrence of noncentrally inserted cords and/or with FGR.

Numerous studies have established [5, 23, 24] a link between abnormal umbilical cord insertion and intrauterine growth restriction and/or low birth weight in all types of multiple pregnancies [47–52]. However, to our knowledge, our study is the first to demonstrate with an accurate characterization of individual newborns growth potential that noncentral cord insertion (i.e., paracentral, battledore, and velamentous) is highly associated with fetal growth restriction and low birth weight in terms of singletons. This result suggests that central UC is relevant to optimal individual growth achievement. In relation to the correlation between peripheral cord insertion and the higher occurrence of FGR, one can speculate from a mathematical and geometrical point of view that a placenta with a central umbilical cord will better ensure an equal distribution and exchange of blood between the different parts of the placenta and directly benefit the growing fetus.

Our study has several limitations. First, we have excluded 75/603 (12.4%) files, which seems high and may provoke concerns regarding biased data. Second, the number of patients was relatively small, which may cover weaker associations such as tobacco/peripheral UC insertion site. Evaluating links between peripheral UC insertion site and rare risk factors will require larger studies. Third, we did not have postpartum follow-up data regarding the growth and the development of children with peripheral UC insertion site. Our results confirmed that it could be relevant to include peripheral UC together with other placental lesions in future epidemiologic studies.

The strength of our study is that the fetal growth was evaluated with appropriate assessment of the newborn personal growth potential. Thanks to AUDIPOG modeling, the characterization of individual estimated percentile allows us to discriminate “constitutionally small” newborns from the ones who failed to achieve their “genetic growth potential”* in utero*.

We have chosen the AUDIPOG module for its specificity to the French population and its continuous updating. We agree that the main nonpathological factors affecting birth weight not only are associated with the maternal age, gestational age, maternal prepregnancy BMI, and parity, but also could be due to ethnic group and paternal characteristics [1]. However, to our knowledge, no module taking into account the ethnic and paternal characteristics has yet been reported for the French population.

Our intent in this study was to evaluate an easily measured criterion—the umbilical cord insertion site at delivery—that would be practical for routine clinical practice. Our results confirm that peripheral UC insertion site should warn obstetrician/paediatrician of a higher risk of FGR and fear its associated adverse perinatal and adult conditions, ranging from stillbirth to chronic diseases in adulthood [3–5]. Therefore, abnormal UC insertion site should be considered as an indication of intensive medical supervision during childhood and adulthood in order to improve FGR associated morbidity and mortality. Furthermore, our results confirm that early prenatal identification of abnormal insertion of the umbilical cord is a desirable clinical goal since these pregnancies are at greater risk for adverse perinatal outcome including not only low birth weight and growth restriction, but also preterm delivery, stillbirth, increased rate of emergency caesarean section, and low Apgar scores [7–12]. As visualization of the placental CI site becomes more difficult with advancing gestation, it should be evaluated in the midtrimester in order to identify a significant number of pregnancies at risk for obstetric complications [53, 54]. The difficulty to image the CI site should warn sonography practitioners of an abnormal CI risk (ultrasound diagnosis and management of umbilical cord abnormalities) and convince them to perform a more precise scan (i.e., scanning in different body positions and using color Doppler). In future, the use of color Doppler ultrasound should be the modality of choice to image the placental cord insertion site at routine obstetric ultrasound, as it allows identifying the placental cord insertion site in practically 100% of cases [53, 54]. Further studies are necessary in order to investigate whether the prenatal diagnosis of an abnormal umbilical cord insertion site in apparent normal fetuses identifies the ones at risk for being growth restricted later in pregnancy and/or at delivery.

## 5. Conclusion 

In conclusion, our results demonstrate a strong link between the umbilical cord insertion site and the optimal individual intrauterine growth potential achievement. We observed that tobacco use was twice as frequent in the peripheral inserted cord group compared to the normal one, though no significant interaction could be reached neither with peripheral umbilical cord insertion site nor with FGR. Extensive studies are required to better understand the physiopathological mechanisms involved in the partial regression of the chorionic villi that define the insertion of the umbilical cord within the placenta. Moreover, we propose that peripheral umbilical cord insertion diagnosis during pregnancy should warn sonography practitioners as this parameter might help in detecting an increased risk of FGR population. Therefore, antenatal ultrasound diagnosis of umbilical cord abnormalities should be considered as an indication of intensive fetal monitoring during pregnancy and labor in order to improve neonatal outcome.

## Figures and Tables

**Table 1:** Types of placentas.

Types of placentas	Central	Peripheral		
		Paracentral	Battledore	Velamentous
Number (%)	343/528 (65.0)	136/528 (25.8)	44/528 (8.3)	5/528 (0.95)
95% CI	[60–70]	[21–33]	[5–16]	[0–9]

**Table 2:** Main demographic characteristics of the mother in the two categories of umbilical cord insertion site.

Variables		Central (*n* = 343)	Peripheral (*n* = 185)	*P* value
Age in years	Mean ± SD	29.4 ± 5.4	29.2 ± 5.3	ns
	Min–max	17–42	16–41	

Parity	Median score	2	2	ns
	[Interquartile range]	[1]	[2]	
	Min–max	1–7	1–8	

Body mass index (BMI) in kg/m^2^	Mean ± SD	22.9 ± 4.2	23.1 ± 4.8	ns
	Min–max	16–41	16–49	

Primiparas	Number (%)	136/343 (39.7%)	81/185 (43.8%)	ns
	95% CI	[0.34–0.45]	[0.37–0.51]	
Multiparas	Number (%)	207/343 (60.3%)	104/185 (56.2%)	ns
	95% CI	[0.55–0.66]	[0.49–2.03]	

Caucasian	Number (%)	222/343 (64.7%)	121/185 (65.4%)	ns
	95% CI	[0.60–0.70]	[0.59–0.72]	
North African	Number (%)	102/343 (29.7%)	55/185 (29.7%)	ns
	95% CI	[0.25–0.35]	[0.23–0.36]	
African	Number (%)	13/343 (3.8%)	8/185 (4.3%)	ns
	95% CI	[0.02–0.06]	[0.01–0.77]	
Asian	Number (%)	6/343 (1.8%)	1/185 (0.6%)	ns
	95% CI	[0.00–0.03]	[−0.01-0.02]	

**Table 3:** Confounding variables involved in fetal growth restriction.

Variables		Central (*n* = 343)	Peripheral (*n* = 185)	*P* value
Prepregnancy pathology	Number (%)	9/343 (2.6%)	7/185 (3.8%)	ns
	95% CI	[0.01–0.04]	[0.01–0.07]	

Uterine anomaly	Number (%)	4/343 (1.2%)	0/185 (0%)	ns
	95% CI	[0.00–0.02]	[0.00-0.00]	

Pregnancy pathology	Number (%)	37/343 (10.8%)	17/185 (9.2%)	ns
	95% CI	[0.08–0.14]	[0.05–0.13]	

Tobacco use	Number (%)	34/343 (9.9%)	37/185 (20.0%)	**0.0012**
	95% CI	[0.07–0.13]	[0.14–0.26]	

**Table 4:** Newborn characteristics in the two categories of umbilical cord insertion site.

Variables		Central (*n* = 343)	Peripheral (*n* = 185)	*P* value
Gestational age at birth in days (mean ± SD)		278.7 (±7.8)	277.4 (±8.4)	ns

Birth weight in grams (mean ± SD)		3433.7 (±376.7)	3195 (±460.5)	**<0.001**

<3rd percentile	Number (%)	5/343 (1.5%)	15/185 (8.1%)	**<0.001**
	95% CI	[0.002–0.028]	[0.042–0.120]	
≥3rd and <10th percentile	Number (%)	12/343 (3.5%)	22/185 (11.9%)	
	95% CI	[0.016–0.054]	[0.072–0.166]	
≥10th and <90th percentile	Number (%)	300/343 (87.5%)	137/185 (74.1%)	
	95% CI	[0.840–0.910]	[0.678–0.804]	
≥90th percentile	Number (%)	26/343 (7.5%)	11/185 (5.9%)	
	95% CI	[0.047–0.103]	[0.025–0.093]	

Growth restricted for gestational age (<10th percentile)	Number (%)	17/343 (4.96%)	37/185 (20.00%)	**<0.001**
	95% CI	[0.027–0.073]	[0.142–0.258]	
Appropriate growth for gestational age	Number (%)	300/343 (87.46%)	137/185 (74.05%)	
	95% CI	[0.840–0.910]	[0.677–0.804]	
Large for gestational age (≥90th percentile)	Number (%)	26/343 (7.58%)	11/185 (5.95%)	
	95% CI	[0.048–0.104]	[0.025–0.094]	

**Table 5:** Adjusted odds ratios (OR) and 95% confidence intervals (95% CI) of peripheral umbilical cord insertion site, tobacco use, and fetal growth restriction using logistic regression.

Variables	OR	95% CI	Coefficient	Standard error	*P* value
Peripheral cord	4.49	[2.26–8.89]	1.50	0.34	**<0.05**
Tobacco use	2.03	[0.55–7.48]	0.71	0.66	ns
Interaction	0.97	[0.20–4.53]	−0.02	0.78	ns

## References

[1] Gardosi J, Chang A, Kalyan B, Sahota D, Symonds EM (1992). Customised antenatal growth charts. *The Lancet*.

[2] Pollack RN, Divon MY (1992). Intrauterine growth retardation: definition, classification, and etiology. *Clinical Obstetrics and Gynecology*.

[3] Mcintire DD, Bloom SL, Casey BM, Leveno KJ (1999). Birth weight in relation to morbidity and mortality among newborn infants. *The New England Journal of Medicine*.

[4] Pilliod RA, Cheng YW, Snowden JM, Doss AE, Caughey AB (2012). The risk of intrauterine fetal death in the small-for-gestational-age fetus. *American Journal of Obstetrics & Gynecology*.

[5] Barker DJP (2006). Adult consequences of fetal growth restriction. *Clinical Obstetrics and Gynecology*.

[6] Redline RW, Kay HH, Nelson MD, Wang Y (2011). The umbilical cord. *The Placenta from Development to Disease*.

[7] Tantbirojn P, Saleemuddin A, Sirois K (2009). Gross abnormalities of the umbilical cord: related placental histology and clinical significance. *Placenta*.

[8] Robinson LK, Jones KL, Benirschke K (1983). The nature of structural defects associated with velamentous and marginal insertion of the umbilical cord. *American Journal of Obstetrics & Gynecology*.

[9] Liu CC, Pretorius DH, Scioscia AL, Hull AD (2002). Sonographic prenatal diagnosis of marginal placental cord insertion: clinical importance. *Journal of Ultrasound in Medicine*.

[10] Raisanen S, Georgiadis L, Harju M, Keski-Nisula L, Heinonen S (2012). Risk factors and adverse pregnancy outcomes among births affected by velamentous umbilical cord insertion: a retrospective population-based register study. *European Journal of Obstetrics & Gynecology and Reproductive Biology*.

[11] Pinar H, Carpenter M (2010). Placenta and umbilical cord abnormalities seen with stillbirth. *Clinical Obstetrics and Gynecology*.

[12] Redline RW (2004). Clinical and pathological umbilical cord abnormalities in fetal thrombotic vasculopathy. *Human Pathology*.

[13] McLennan JE (1968). Implications of the eccentricity of the human umbilical cord. *American Journal of Obstetrics & Gynecology*.

[14] Akpom PU, Fox H (1977). The clinical significance of marginal and velamentous insertion of the cord. *British Journal of Obstetrics and Gynaecology*.

[15] Gavriil P, Jauniaux E, Leroy F (1993). Pathologic examination of placentas from singleton and twin pregnancies obtained after in vitro fertilization and embryo transfer. *Pediatric Pathology*.

[16] Luo G, Redline RW (2013). Peripheral insertion of umbilical cord. *Pediatric and Developmental Pathology*.

[17] Benirschke K, Kaufmann P (2000). *Pathology of the Human Placenta*.

[18] Hertig AT (1967). Human trophoblast: normal and abnormal. A plea for the study of the normal so as to understand the abnormal. Ward Burdick Award Address. *American Journal of Clinical Pathology*.

[19] Nordenvall M, Sandstedt B, Ulmsten U (1988). Relationship between placental shpae, cord insertion, lobes and gestational outcome. *Acta Obstetricia et Gynecologica Scandinavica*.

[20] Salafia CM, Yampolsky M, Shlakhter A, Mandel DH, Schwartz N (2012). Variety in placental shape: when does it originate?. *Placenta*.

[21] DiSalvo D (1998). The correlation between placental pathology and intraventricular hemorrhage in the preterm infant. The developmental epidemiology network investigators. *Pediatric Research*.

[22] Benirschke K, Kaufmann P, Baergen R (2006). Anatomy and pathology of the umbilical cord. *Pathology of the Human Placenta*.

[23] Ebbing C, Kiserud T, Johnsen SL, Albrechtsen S, Rasmussen S (2013). Prevalence, risk factors and outcomes of velamentous and marginal cord insertions: a population-based study of 634,741 pregnancies. *PLoS ONE*.

[24] Misra DP, Salafia CM, Miller RK, Charles AK (2009). Non-linear and gender-specific relationships among placental growth measures and the fetoplacental weight ratio. *Placenta*.

[25] Burton GJ, Jauniaux E (2004). Placental oxidative stress: from miscarriage to preeclampsia. *Journal of the Society for Gynecologic Investigation*.

[26] Burton GJ, Jauniaux E, Watson AL (1999). Maternal arterial connections to the placental intervillous space during the first trimester of human pregnancy: the Boyd collection revisited. *American Journal of Obstetrics & Gynecology*.

[27] Mamelle N, Munoz F, Grandjean H (1996). Fetal growth from the AUDIPOG study. I. Establishment of reference curves. *Journal de Gynécologie, Obstétrique et Biologie de la Reproduction*.

[28] Mamelle N, Munoz F, Martin JL, Laumon B, Grandjean H (1996). Fetal growth from the AUDIPOG study. II. Application for the diagnosis of intrauterine growth retardation. *Journal de Gynécologie, Obstétrique et Biologie de la Reproduction*.

[29] Mamelle N, Boniol M, Rivière O (2006). Identification of newborns with Fetal Growth Restriction (FGR) in weight and/or length based on constitutional growth potential. *European Journal of Pediatrics*.

[30] Mamelle N, Cochet V, Claris O (2001). Definition of fetal growth restriction according to constitutional growth potential. *Biology of the Neonate*.

[31] Rolschau J (1978). The relationship between some disorders of the umbilical cord and intrauterine growth retardation. *Acta Obstetricia et Gynecologica Scandinavica*.

[32] Prada JA, Tsang RC (1998). Biological mechanisms of environmentally induced causes of IUGR. *European Journal of Clinical Nutrition*.

[33] Sankaran S, Kyle PM (2009). Aetiology and pathogenesis of IUGR. *Best Practice and Research: Clinical Obstetrics and Gynaecology*.

[34] Montan S (2007). Increased risk in the elderly parturient. *Current Opinion in Obstetrics and Gynecology*.

[35] Koo YJ, Ryu HM, Yang JH (2012). Pregnancy outcomes according to increasing maternal age. *Taiwanese Journal of Obstetrics and Gynecology*.

[36] Romo A, Carceller R, Tobajas J (2009). Intrauterine growth retardation (IUGR): epidemiology and etiology. *Pediatric Endocrinology Reviews*.

[37] Ergaz Z, Avgil M, Ornoy A (2005). Intrauterine growth restriction—etiology and consequences: what do we know about the human situation and experimental animal models?. *Reproductive Toxicology*.

[38] Reeves S, Bernstein I (2008). Effects of maternal tobacco-smoke exposure on fetal growth and neonatal size. *Expert Review of Obstetrics and Gynecology*.

[39] D’Angelo DV, Whitehead N, Helms K, Barfield W, Ahluwalia IB (2011). Birth outcomes of intended pregnancies among women who used assisted reproductive technology, ovulation stimulation, or no treatment. *Fertility and Sterility*.

[40] Meeker JD (2012). Exposure to environmental endocrine disruptors and child development. *Archives of Pediatrics & Adolescent Medicine*.

[41] DiVall SA (2013). The influence of endocrine disruptors on growth and development of children. *Current Opinion in Endocrinology, Diabetes & Obesity*.

[42] Smarr MM, Vadillo-Ortega F, Castillo-Castrejon M, O'Neill MS (2013). The use of ultrasound measurements in environmental epidemiological studies of air pollution and fetal growth. *Current Opinion in Pediatrics*.

[43] Thirkill TL, Vedagiri H, Douglas GC (2006). Macaque trophoblast migration toward RANTES is inhibited by cigarette smoke-conditioned medium. *Toxicological Sciences*.

[44] Holloway AC, Salomon A, Soares MJ (2014). Characterization of the adverse effects of nicotine on placental development: in vivo and in vitro studies. *American Journal of Physiology: Endocrinology and Metabolism*.

[45] Shiverick KT, Salafia C (1999). Cigarette smoking and pregnancy. I: ovarian, uterine and placental effects. *Placenta*.

[46] Zdravkovic T, Genbacev O, Prakobphol A (2006). Nicotine downregulates the l-selectin system that mediates cytotrophoblast emigration from cell columns and attachment to the uterine wall. *Reproductive Toxicology*.

[47] Cai L-Y, Izumi S-I, Koido S (2006). Abnormal placental cord insertion may induce intrauterine growth restriction in IVF-twin pregnancies. *Human Reproduction*.

[48] Kent EM, Breathnach FM, Gillan JE (2011). Placental cord insertion and birthweight discordance in twin pregnancies: results of the national prospective ESPRiT study. *American Journal of Obstetrics & Gynecology*.

[49] Loos RJF, Derom C, Derom R, Vlietinck R (2001). Birthweight in liveborn twins: the influence of the umbilical cord insertion and fusion of placentas. *British Journal of Obstetrics and Gynaecology*.

[50] de Paepe ME, Shapiro S, Young L, Luks FI (2010). Placental characteristics of selective birth weight discordance in diamniotic-monochorionic twin gestations. *Placenta*.

[51] Hanley ML, Ananth CV, Shen-Schwarz S, Smulian JC, Lai Y-L, Vintzileos AM (2002). Placental cord insertion and birth weight discordancy in twin gestations. *Obstetrics and Gynecology*.

[52] Baxi LV, George EM (2013). Three-way tie: the umbilical cord insertion site was different for each member of a set of triplets. *American Journal of Obstetrics & Gynecology*.

[53] Nomiyama M, Toyota V, Kawano H (1998). Antenatal diagnosis of velamentous umbilical cord insertion and vasa previa with color Doppler imaging. *Ultrasound in Obstetrics and Gynecology*.

[54] Sepulveda W, Rojas I, Robert JA, Schnapp C, Alcalde JL (2003). Prenatal detection of velamentous insertion of the umbilical cord: a prospective color Doppler ultrasound study. *Ultrasound in Obstetrics and Gynecology*.

